# Smad2 Preserves Corneal Stromal Homeostasis by Restraining Profibrotic Smad3/YAP/TEAD2 Transcriptional Program

**DOI:** 10.3390/cells15131202

**Published:** 2026-07-02

**Authors:** Ruimei Zhou, Dunpeng Cai, Shi-You Chen

**Affiliations:** 1 Departments of Surgery, University of Missouri School of Medicine, Columbia, MO 65212, USA; rz8bx@missouri.edu (R.Z.);; 2 Departments of Medical Pharmacology and Physiology, University of Missouri School of Medicine, Columbia, MO 65212, USA; 3 The Research Service, Harry S. Truman Memorial Veterans Hospital, Columbia, MO 65201, USA

**Keywords:** Smad2, corneal stromal homeostasis, corneal opacity, TGF-β/Smad3 signaling, YAP/TEAD2

## Abstract

Corneal transparency depends on quiescence of stromal cells derived from neural crest cells and a well-controlled extracellular matrix. Disruption of this homeostasis causes fibrotic scarring, a leading cause of blindness. Transforming growth factor-β/Smad3 signaling drives corneal fibrogenesis, but the distinct roles of Smad2 versus Smad3 remain unclear. Smad2 ablation in neural crest cells using Wnt1-Cre mice triggers spontaneous severe corneal opacification along with massive stromal hypercellularity and fibrosis. The fibrotic phenotype occurs in the absence of injury, indicating that Smad2 is essential for balancing Smad3 activity in driving fibrotic signaling. Single-cell RNA sequencing and virtual knockout of Smad2 reveal prominent activation of Smad3-Yes-associated protein (YAP)/TEAD2-transcriptional program in Smad2-null corneas. Biochemical assays confirm that Smad2 loss results in increased Smad3 phosphorylation and formation of nuclear Smad3–YAP–TEAD2 complex. This trimeric complex induces the expression of collagen I, connective tissue growth factor, and cyclin D1. Importantly, pharmacologic inhibition of YAP/TEAD interaction with verteporfin blocks stromal hyperplasia and corneal fibrosis by suppressing the expression of fibrotic and cell cycle genes, which lead to restoration of corneal transparency in Smad2-neural crest-deficient mice. Our findings reveal a unique convergence of YAP/TEAD and TGF-β/Smad3 signaling that can be targeted with verteporfin to prevent corneal scarring and blindness.

## 1. Introduction

The cornea is an avascular and transparent tissue that provides most of the refractive power and serves as a protective barrier to eye [1]. Optical clarity is ensured by the precise architecture of the corneal stroma. The highly ordered collagen fibrils (primarily Type I and V) interwoven with proteoglycans form a lattice that minimizes light scattering [2]. This structure is maintained by corneal stromal cells (CSCs), a quiescent network of neural-crest-derived cells residing between collagen lamellae [3]. Under normal conditions, CSCs adopt a dendritic morphology and a low metabolic and contact-inhibited state dedicated to slow turnover of the extracellular matrix (ECM). Notably, this quiescence is an actively regulated state, despite constant mild stimuli such as intraocular pressure fluctuations, blinking-induced shear stress, and basal tear film cytokines. Healthy CSCs remain phenotypically silent to preserve transparency [4].

Corneal homeostasis can be disrupted by injury or inflammation, and a rapid wound healing cascade is then triggered [5]. Quiescent CSCs lose their dendritic property to become proliferative and/or differentiate into fibroblasts. Under persistent inflammatory or transforming growth factor-β (TGF-β) signaling, these fibroblasts further transdifferentiate into α-smooth muscle actin (α-SMA)–expressing myofibroblasts [6]. While this response is crucial for healing wounds and preventing globe rupture, it comes at the cost of optical clarity. Activated myofibroblasts secrete disordered, opaque ECM components, especially collagens (Type I and III) and connective tissue growth factor (CTGF), and exert contractile forces on the stroma [7]. The resulting scar tissue or corneal “haze” damages vision and can lead to irreversible blindness, often requiring surgical intervention. Previous injury models (e.g., blast or chemical injuries) have established that aberrant TGF-β signaling is the key regulator of this fibrotic cascade in the cornea [8].

TGF-β is a pleiotropic cytokine that governs cell proliferation, differentiation, and ECM remodeling in virtually all tissues. In the injured cornea, latent TGF-β1 and TGF-β2 are released from the epithelium, tear film, and infiltrating immune cells [8]. Canonically, TGF-β ligands bind a heterotetrameric receptor complex of type II and type I serine/threonine kinase receptors on the target cell surface [9]. Ligand engagement causes the constitutive TGF-βRII to phosphorylate and activate TGF-βRI, and the activated TGF-βRI then propagates the signal to downstream Smads, i.e., phosphorylating Smad2 and Smad3, at their C-terminal SSXS motifs [10]. Phosphorylated Smad2/3 dissociates from the receptor and forms a heterotrimeric complex with co-mediator Smad4, which translocates into nuclei to regulate gene transcription. In the nuclei, Smad complexes bind specific Smad-binding elements (CAGA-box sequences) in target gene promoters, driving the expression of fibrosis-related genes [11]. TGF-β receptors can also activate Smad-independent pathways (e.g., MAPK, PI3K/AKT, Rho/ROCK) that synergize with Smad signaling to reinforce myofibroblast differentiation [12]. Historically, Smad2 and Smad3 are thought to be functionally redundant, owing to their high sequence similarity and shared activation mechanism. However, accumulating evidence indicates that Smad3 is the primary transcriptional driver of pathological fibrosis, whereas Smad2 performs distinct roles in development and homeostasis [13,14,15]. Genetic studies have shown that Smad3 deletion attenuates fibrosis in multiple organs [16], while Smad2 deletion leads to early embryonic lethality and disrupts neural crest development [17], suggesting non-redundant functions. The structural basis for these divergent roles lies in a unique 30 amino acid insertion (the “E3 insert”) in Smad2’s DNA-binding MH1 domain that is absent in Smad3. This small insert sterically hinders Smad2 from binding DNA directly [18,19]. Consequently, Smad2/Smad4 complexes must recruit additional cell-specific co-factors (e.g., FOXH1) to bind promoters [18], whereas Smad3/Smad4 complexes can directly recognize CAGA elements and robustly activate fibrotic gene promoters [19]. Because Smad2 and Smad3 share same upstream activators and the co-Smad partner, they inherently compete for receptor binding and the limited pool of Smad4 in cells. This competition may make Smad2 an effective restraint on Smad3 signaling by sequestering key signaling components away from Smad3 [15,20]. In essence, abundant Smad2 can occupy TGF-β receptors and Smad4, channeling signaling into Smad2-dependent (often developmentally essential) programs while dampening Smad3-driven pro-fibrotic transcription. Such a mechanism would buffer the cornea against inappropriate fibrogenesis in response to minor TGF-β fluctuations, thereby maintaining the CSC’s quiescent, transparency-preserving state [15].

In parallel, corneal stromal cells are profoundly influenced by biomechanical cues from their ECM. The principal pathway transducing mechanical and density signals is the Hippo–YAP/TAZ pathway. Under conditions of high cell density or a soft matrix (physiological conditions in a healthy cornea), the Hippo core kinases MST1/2 and LATS1/2 are active and phosphorylate the transcriptional co-activators YAP (Yes-associated protein) and TAZ, anchoring them in the cytoplasm and targeting them for degradation [21]. Conversely, when cells experience low density, increased matrix stiffness, or certain extracellular signals, Hippo signaling is turned “off,” allowing unphosphorylated YAP/TAZ to accumulate in the nucleus [21]. Nuclear YAP/TAZ lack DNA-binding capacity on their own and thus must partner with DNA-binding transcription factors, predominantly the TEAD family (i.e., TEAD1–4), to modulate gene expression [22]. YAP/TAZ–TEAD complexes activate a broad transcriptional program that promotes cell proliferation, survival, and ECM production, all of which are hallmarks of fibrotic stromal activation. Among the TEAD proteins, TEAD1 and TEAD4 are the dominant mediators of YAP-driven gene expression in adult tissues [23]. In contrast, TEAD2 is most active during development (e.g., neural tube closure, stem cell maintenance) and is largely silenced in adult tissues under homeostasis. Intriguingly, TEAD2 often resurfaces in pathological contexts such as epithelial–mesenchymal transition (EMT) and carcinogenesis, suggesting that it may be specifically deployed during major cellular reprogramming or dedifferentiation [24].

Recent studies have revealed crosstalk between TGF-β/Smad and Hippo/YAP signaling, suggesting that biochemical and mechanical signals cooperate to drive myofibroblast activation [25,26,27,28,29,30]. YAP has been shown to physically interact with Smad proteins, influencing their nuclear localization and target gene selection [31]. However, the precise integration of these pathways in the cornea and in particular how Smad2 versus Smad3 might differentially modulate the YAP/TEAD-driven transcriptional program remain poorly understood. This gap is especially relevant given the cornea’s unique developmental origin and the requirement for strict homeostatic control. The anterior segment of the eye (including the corneal stroma and endothelium) is derived from cranial neural crest cells, which migrate to the developing eye and differentiate under tight spatiotemporal regulation of TGF-β and Hippo pathways [32]. Studying gene function in this neural-crest-derived tissue is challenging, as global knockout of essential genes like Smad2 causes early embryonic lethality. To overcome this, we employed a Wnt1-Cre transgenic strategy to delete Smad2 specifically in neural-crest-derived cells [33]. The Wnt1 promoter is active during early neural crest development [34], enabling Cre-mediated recombination of floxed Smad2 alleles in CSCs (and other neural crest lineages) while sparing Smad2 expression in non-neural crest tissues. This approach allowed us to investigate the cell-intrinsic role of Smad2 in maintaining adult corneal transparency and stromal quiescence.

Here, we report that Smad2 is indispensable for preserving corneal clarity and CSC quiescence. Conditional loss of Smad2 in neural-crest-derived cells leads to spontaneous corneal opacity and stromal hyperplasia, demonstrating that the corneal stroma initiates a fibrotic response even in the absence of external injury. Through single-cell transcriptomics and molecular analyses, we find that Smad2 suppresses a latent profibrotic program orchestrated by phosphorylated Smad3, YAP, and TEAD2. In Smad2-deficient corneas, unchecked TGF-β signaling via Smad3 synergizes with aberrant YAP/TEAD2 activity to drive excessive CSC proliferation and ECM deposition. Notably, pharmacological disruption of YAP–TEAD interaction effectively halts this pathological program and restores corneal transparency.

## 2. Methods

### 2.1. Mouse Strains and Conditional Knockout Generation

All animals were housed in conventional conditions, and all animal experiments were performed in compliance with Institutional Animal Care and Use Committee guidelines of the University of Missouri. Wnt1-Cre; Smad2fl/fl mice were generated by crossing transgenic Wnt1-Cre mice (The Jackson Laboratories, 022137) with Smad2 floxed mice. A Rosa26-loxSTOP-lox-eYFP reporter (R26R-EYFP) was also introduced to label neural crest cell lineage. Adult Wnt1-Cre; Smad2fl/fl; R26R-EYFP mice (Smad2nc-/-) and Wnt1-Cre; R26R-EYFP littermate controls (16 mice each group) were used in the study based on prior study experience. The incidence of the corneal phenotype was recorded at 4 months old through slit-lamp examination, and eyes were photographed under a stereomicroscope.

### 2.2. Sex as a Biological Variable

Both male and female mice (8–12 weeks old) were included in in vivo experiments, and animals were distributed across control and experimental groups without intentional exclusion of either sex. Because sex-stratified analyses of the corneal phenotype did not show differences between male and female mice, the subsequent experiments were conducted with samples from equal numbers of male and female mice.

### 2.3. Corneal Tissue Processing and Histology

Eyes were harvested and immediately fixed in 4% paraformaldehyde (PFA) in PBS at 4 °C for 24 h. Tissues were washed and then processed for paraffin embedding and sectioning at 5 μm thickness. For hematoxylin and eosin (H&E) staining, paraffin sections were deparaffinized in xylene, rehydrated through a graded ethanol series, and rinsed in distilled water. Sections were stained with hematoxylin, rinsed in running tap water, differentiated and blued as needed, and then counterstained with eosin. After staining, sections were dehydrated through graded ethanol, cleared in xylene, and mounted with a permanent mounting medium. Images of whole-eye and corneal regions were acquired using a bright-field microscope. Corneal stromal thickness and cellularity were quantified from digitized H&E images using ImageJ (version 1.54, National Institutes of Health, Bethesda, MD, USA).

To assess stromal fibrosis and collagen deposition, adjacent paraffin sections were subjected to Picrosirius Red staining using an Abcam staining kit (ab246832, Cambridge, UK) according to the manufacturer’s instructions. Briefly, sections were deparaffinized, rehydrated, stained with Picrosirius Red solution, washed in acidified water, dehydrated, and mounted for imaging. Collagen accumulation in the corneal stroma was evaluated from brightfield images and quantified using ImageJ. Three mid-cornea regions per eye were measured and averaged per animal.

### 2.4. Immunostaining and EdU Proliferation Assay

For lineage tracing and proliferation analyses, 5-ethynyl-2′-deoxyuridine (EdU) labeling was performed in vivo. Control and Smad2nc-/- mice were intraperitoneally injected with EdU (100 mg/kg) 2 h before euthanasia. Eyes were fixed in 4% PFA for 12 h, cryoprotected in 30% sucrose, and cryosectioned at 5 μm. Sections were permeabilized with 0.3% Triton X-100, and EdU incorporation was detected with a Click-iT Alexa Fluor 594 kit (Thermo Scientific, Waltham, MA, USA) following the manufacturer’s protocol. For immunostaining, sections were blocked in 5% normal goat serum and incubated with primary antibodies overnight at 4 °C. Neural-crest-derived cells were identified using an eYFP reporter. Primary antibodies included rabbit anti-GFP (1:1000, Aves, Davis, CA, USA, to amplify EYFP) and others as specified below. After washing, slides were incubated with species-appropriate Alexa-Fluor-conjugated secondary antibodies (1:5000, Life Technologies, Carlsbad, CA, USA) for 1 h at room temperature. Nuclei were counterstained with DAPI (1 μg/mL). Stained sections were mounted in ProLong Gold (Invitrogen, Carlsbad, CA, USA) and imaged with a Nikon C2 laser-scanning confocal microscope. For EdU quantification, at least five non-overlapping fields in central stroma were imaged per cornea. The percentage of EdU+ nuclei among EYFP+ (neural crest) cells was calculated for each sample.

### 2.5. Single-Cell RNA Sequencing Analysis and In Silico Knockout

To profile corneal cell populations, we analyzed a published single-cell RNA sequencing dataset of the adult mouse cornea (NCBI BioProject PRJNA891516) [35]. Raw sequencing data were processed with the Seurat (v4) pipeline: cells with low UMI counts or high mitochondrial read fraction were filtered, and expression matrices were log-normalized. We performed principal component analysis (PCA) on the top variable genes and identified clusters using the Louvin algorithm at moderate resolution. Clusters were annotated based on established marker genes, e.g., Kera for stromal keratocytes, Col8a1 for corneal endothelium, and Krt12 for epithelium. UMAP visualization confirmed distinct cell populations, and FeaturePlots/ViolinPlots showed that Smad2 was highly expressed in stromal keratocytes, fibroblasts, and neural-crest-derived mesenchyme.

For network analysis, we applied the scTenifoldKnk algorithm to perform an in silico Smad2 knockout in the stromal keratocyte subset [36]. Briefly, scTenifoldKnk constructs a gene regulatory network (GRN) from wild-type single-cell data and then computationally “deletes” the target gene (Smad2) from the network. We built a baseline CSC GRN using tensor decomposition of gene covariance and then removed Smad2, aligning the perturbed GRN to the original one via manifold alignment. Genes with the largest network destabilization (high “perturbation scores”) in the virtual knockout were identified as the potential candidate effectors of Smad2 loss. Subsequent experiments were designed to test whether the predicted candidates are regulated and associated with the fibrotic phenotype in Smad2nc-/- corneas.

### 2.6. Co-Immunoprecipitation and Western Blotting

For protein interaction studies, corneas from control and Smad2nc-/- mice were dissected to isolate the stromal tissue. Approximately 6 corneas per genotype were pooled and homogenized in ice-cold RIPA lysis buffer (50 mM Tris-HCl pH 7.4, 150 mM NaCl, 1% NP-40, 0.5% sodium deoxycholate, 0.1% SDS) with protease/phosphatase inhibitors. Lysates were clarified (14,000× *g*, 15 min), and equal amounts of proteins (500 μg) were incubated overnight at 4 °C with 2 μg of primary antibody, i.e., anti-phospho-Smad3 (p-Smad3, Ser423/425) (Cell Signaling, Danvers, MA, USA, #9520), anti-TEAD2 (Abcam, ab112627), or anti-YAP (Cell Signaling, #14074). Primary antibody information is also included in Supplementary Table S1. Immune complexes were captured with Protein A/G agarose beads (Thermo), washed three times in lysis buffer and eluted by boiling in SDS sample buffer. For Western blot, input lysates and IP eluates were resolved in 4–12% SDS-PAGE gels and transferred to PVDF membranes. Blots were blocked in 5% milk/TBST and probed with the following primary antibodies (overnight at 4 °C): anti-pSmad3 (Cell Signaling, 1:1000), anti-YAP (ThermoFisher, 1:1000), anti-TEAD2 (ThermoFisher, 1:1000), anti-CTGF (Novus, Centennial, CO, USA, 1:1000), anti-COL1A1 (Abcam, 1:1000), anti-Cyclin D1 (Abcam, 1:2000), or anti-PCNA (Cell Signaling, 1:1000) with anti-GAPDH (Cell Signaling, 1:50,000) as the loading control. HRP-conjugated secondary antibodies (ThermoFisher, 1:5000) were applied for 1 h at room temperature. Signals were developed using ECL substrate and captured on a BioRad imaging system (version 6.1, Bio-Rad Laboratories, Hercules, CA, USA). Band intensities were quantified with ImageJ software, and phosphorylated Smad3 levels were normalized to total Smad3 or GAPDH. Co-IP blots were also probed to co-precipitate YAP and TEAD2.

### 2.7. Proximity Ligation Assay (PLA)

To visualize in situ protein–protein interactions within corneal stromal cells, we performed Duolink PLA (Sigma, St. Louis, MO, USA, # DUO92001 & DUO92002) on flat-mounted corneas. Freshly enucleated eyes were fixed in 2% PFA for 30 min, and the corneas were dissected, permeabilized with 0.5% Triton X-100, and blocked in Duolink blocking solution. Samples were incubated with primary antibody pairs overnight at 4 °C: mouse anti-YAP (1:200) paired with rabbit anti-pSmad3 (1:500) for YAP-Smad3 interaction and mouse anti-TEAD2 (1:700) paired with rabbit anti-p-Smad3 or with rabbit anti-YAP for TEAD2-Smad3 or TEAD2–YAP interactions. After washing, corneas were incubated with species-specific PLA secondary probes. Ligation and polymerase rolling-circle amplification were carried out according to the manufacturer’s instructions. After the final washes, tissues were counterstained with DAPI and mounted. Confocal z-stacks were acquired (20× objective), and PLA signals (red, fluorescent puncta) indicating protein interactions were quantified per nucleus. Negative controls (single primary or isotype controls) yielded no puncta.

### 2.8. Luciferase Reporter Assay

Primary corneal stromal cells (keratocytes) were isolated from adult mouse corneas through collagenase digestion and flow cytometry sorting. Briefly, corneas were excised, cut into small pieces, and incubated in DMEM with 2 mg/mL of collagenase I for 1 h at 37 °C. The dissociated cells were filtered and cultured in fibroblast growth medium (DMEM with 10% FBS and 1% penicillin/streptomycin). Low-passage cells from control and Smad2nc-/- mice were used for transient transfections. For pathway activation assays, cells were co-transfected with firefly luciferase reporters and a constitutive Renilla luciferase internal control (pRL-TK, Promega, Madison, WI, USA, #E2231) using Lipofectamine 3000. The reporters used were SBE4-Luc (Addgene plasmid, #16495, a Smad3/4-responsive 4×CAGA Smad binding element (SBE)-driving luciferase) and 8×GTIIC-luc (Addgene, #34615, TEAD/YAP-responsive 8×GTIIC elements). TOPFlash (Wnt/β-catenin reporter, 8× TCF sites) was used as a pathway specificity control. Then, 48 h later, dual-luciferase assays were performed with cell lysates. In some assays, SIS3 (Selective Smad3 inhibitor, 10 μM, Tocris, Bristol, UK) was added 6 h post-transfection, and luciferase was measured at 24 h.

For Col1a1 and Ctgf promoter analyses, we employed custom luciferase reporters. A 2.3 kb fragment of the mouse Col1a1 promoter was cloned into pGL4.10 (Promega). Site-directed mutagenesis was performed to generate versions with the Smad-binding element (SBE) or TEAD-binding element mutated (AGAC to ATAT for SBE; and 5′-CATTCC-3′ to 5′-GGGGGG-3′ for TEAD site). Similarly, a 1.1 kb Ctgf promoter (containing the distal modular region with TEAD sites) was cloned, and a ΔTEAD mutant was created by deleting its three TEAD sites. Keratocytes were transfected with these reporters and other expression plasmids as indicated. To test YAP dependence, we co-transfected constitutively active YAP (5SA) or a TEAD-binding-deficient YAP mutant (5SA/S94A) (YAP 5SA adds Ser-to-Ala mutations at LATS phosphorylation sites; S94A abrogates TEAD interaction).

Luciferase reporter plasmid transfection was optimized to gain at least 10^4^ relative light units for each construct before the actual reporter assays were performed. Cell viability was assessed in parallel using trypan blue staining. No significant reduction in cell viability was observed under the transfection or treatment conditions. All luciferase reporter assays were performed with independent biological replicates of primary corneal stromal cells/keratocytes isolated from control and Smad2nc-/- mice. For each experiment, cells from independent isolations were transfected separately and considered as biological replicates. Firefly luciferase activity was normalized to Renilla luciferase activity. Technical replicate wells were averaged first, and the averaged values were used for statistical analysis.

### 2.9. Pharmacological Inhibition of YAP/TEAD Interaction

To therapeutically target the Smad3–YAP–TEAD axis, verteporfin, a pharmacological inhibitor of YAP–TEAD interaction, was administered topically to the ocular surface of six Smad2nc-/- mice using a viscous gel formulation designed to prolong corneal residence time. Verteporfin (Sigma-Aldrich, #S1786) was first dissolved in DMSO to prepare a concentrated stock solution and then diluted into a sterile hyaluronic-acid-based ophthalmic gel/hydrogel vehicle to achieve a final working concentration of 100 μM. Vehicle control gel was prepared in parallel with the same final concentration of DMSO but without verteporfin. Treatment was initiated on postnatal day 22. For each administration, mice were lightly anesthetized, and approximately 20 μL of verteporfin-containing gel was applied directly onto the central corneal surface of each eye. The gel was administered once daily throughout the treatment period. Six control mice received an equal volume of the matched vehicle gel on the same schedule. Corneal opacity and surface appearance were monitored longitudinally through stereomicroscopy during treatment. Mice were maintained on this regimen until 3 months of age, at which time they were euthanized through CO_2_ inhalation and cervical dislocation, and corneas were collected for downstream histological, molecular, and immunostaining analyses. During verteporfin treatment, mice were monitored for body weight, gross ocular appearance, and signs of ocular irritation or distress. No treatment-related mortality was observed during the study, and a few mice showed body weight loss. Although the cause of this weight loss was not clear, potential contributing factors include the congenital ocular disease burden, repeated topical gel administration, repeated handling/anesthesia, treatment-related stress, or animal to animal variability. Future toxicity assessment would be needed to determine the potential side effects of the treatment, although verteporfin has been used in clinic already.

### 2.10. Randomization and Blinding

For genotype-based comparisons, experimental groups were defined by genotype using littermate controls. Adult mice of comparable age were included, and both sexes were represented. For verteporfin treatment experiments, Smad2nc-/- mice were assigned to vehicle or verteporfin treatment groups randomly. Histological and immunofluorescence images were coded before quantification, and image-based analyses, including corneal thickness, collagen-positive area, eYFP-positive cell percentage, EdU-positive cell percentage, and fluorescence intensity, were performed by an investigator blinded to genotype or treatment group whenever feasible. Because some phenotypes, including gross corneal opacity and marked stromal thickening, were visually apparent, complete blinding during all stages of sample collection, and image acquisition was not always possible.

### 2.11. Statistical Analysis

All quantitative data are presented as mean ± SD. When both eyes from the same animal were analyzed for the same endpoint, values from the two eyes were averaged to generate one value per mouse for statistical analysis. When only one eye was used for histology, immunostaining, or protein analysis, one eye per mouse was included. The number of independent animals used for each analysis is indicated in the corresponding figure legends. Sex difference in corneal phenotype incidences (percentage data) was analyzed using the two-sided Fisher’s exact test. Other data were analyzed using two-tailed Student’s t-tests or one-way ANOVA with post hoc Tukey’s test for multi-group comparisons, as appropriate. *p* < 0.05 was considered statistically significant. Data plotting and statistical analyses were performed using GraphPad Prism 10.

## 3. Results

### 3.1. Loss of Smad2 in Neural Crest Cells Leads to Spontaneous Corneal Opacity and Stromal Thickening

**Figure 1:**
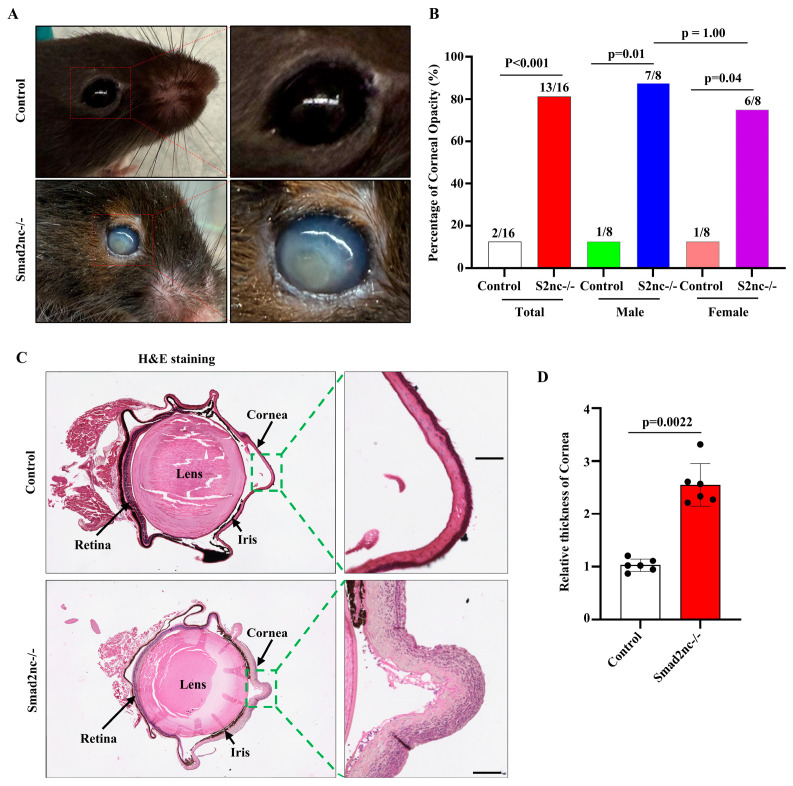
Smad2 deficiency in neural crest cells resulted in spontaneous corneal opacity and stromal hyperplasia. (**A**) Representative gross eye images of adult Wnt1-Cre (Control) and Wnt1-Cre; Smad2fl/fl (Smad2nc-/-) mice. Red dashed boxes indicate the magnified area shown on the right. Smad2nc-/- mutant mice exhibited severe central corneal opacity compared to the clear corneas of the controls. (**B**) Incidence of corneal opacity in control and mutant mice. The severe phenotype was observed in 81.3% (13/16) of Smad2nc-/- mice, while the mild phenotype observed in 2 out of 16 controls. No sex difference was observed in the incidence of corneal opacity, as analyzed using the two-sided Fisher’s exact test. The reported *p* values are unadjusted. (**C**) Representative hematoxylin and eosin (H&E) staining of whole eye sections (left) and the magnified corneal regions in the green dashed box (right). Scale bar: 50 μm. Significant thickening and hypercellularity of the corneal stroma were shown in the mutant group. (**D**) Quantification of relative corneal thicknesses. Data are presented as mean ± SD. Student’s t-test, n = 6.

To investigate the physiological role of Smad2 in corneal homeostasis, we deleted *Smad2* in neural-crest-derived cells. Smad2nc-/- mice exhibited a striking ocular phenotype characterized by severe corneal opacity, appearing as dense white plaques (Figure 1A). This phenotype was highly penetrant, observed in 81.3% (13/16) of Smad2nc-/- mice, while only 12.5% (2/16) of Wnt1-Cre control mice exhibited mild corneal abnormalities (Figure 1B), likely due to Wnt1-Cre-related adverse effects. To determine if there is a sex difference in Smad2nc-/-caused phenotype, we analyzed the incidence of corneal opacity in both male and female mice and found that Smad2nc-/- caused similar defective corneas in male (7/8) and female mice (6/8), indicating that Smad2 is equally essential for protecting eyes of both male and female animals. Because there is no sex difference in Smad2nc-/- caused corneal opacity, we used samples from equal numbers of male and female mice for all the subsequent experiments/analyses.

Histological analysis via H&E staining revealed profound alterations in the corneal architecture of Smad2nc-/- mice. While control corneas maintained a thin and organized stroma, Smad2nc-/- corneas displayed significant stromal thickening and hypercellularity (Figure 1C). Quantification confirmed a greater than two-fold increase in relative corneal thickness in the Smad2nc-/- group (Figure 1D). These data indicate that *Smad2* is indispensable for maintaining corneal transparency and stromal homeostasis.

### 3.2. Smad2-Deficient Cornea Thickening Is Driven by Aberrant Proliferation of Neural Crest-Derived Cells

**Figure 2:**
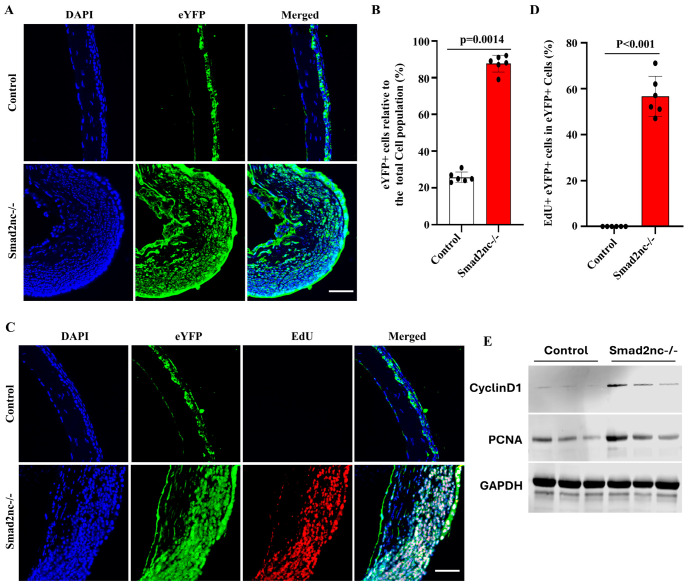
Smad2 deficiency triggered aberrant proliferation of neural-crest-derived corneal stromal cells. (**A**) Representative immunostaining of corneal sections from Wnt1-Cre; eYFP (Control) and Wnt1-Cre; eYFP; Smad2fl/fl (Smad2nc-/-) mice. Nuclei were stained with DAPI (blue) and neural-crest-derived cells were marked by eYFP (green), which was also present in control cornea, due to the Wnt1-Cre-caused labeling of neural crest lineage (also in panel (**C**)). Note the massive expansion of the eYFP+ stromal layer in the corneas of the mutant mice. Scale bar: 25 µm. (**B**) The percentage of eYFP+ cells relative to total cell population in the corneas, n = 6, Student’s t-test. (**C**) Cell proliferation as assessed by EdU incorporation assay. EdU incorporation (red) in eYFP+ cells (green) indicated proliferation of neural-crest-derived cells. Control corneas showed cellular quiescence, while corneas of Smad2nc-/- mice exhibited extensive proliferation in eYFP+ cell populations. Nuclei were stained with DAPI (blue). Scale bar: 25 µm. (**D**) Quantification of cell proliferation rate, shown as the percentage of EdU+ cells relative to the total eYFP+ cell population. Data are presented as mean ± SD. n = 6, Student’s t-test. (**E**) Representative Western blot analysis of cell cycle regulators. The protein levels of Cyclin D1 and PCNA were markedly upregulated in Smad2nc-/- corneas compared to the control group, indicating that the proliferative phenotype was driven by enhanced cell cycle machinery. GAPDH is a loading control.

To determine if the observed stromal thickening was due to the expansion of neural-crest-derived populations, we tracked neural crest lineage using *Rosa26*-eYFP reporter with Wnt1-Cre-mediated recombination, which revealed a massive expansion of the eYFP+ stromal layer in Smad2nc-/- corneas compared to the thin layer in control corneas (Figure 2A). The proportion of neural-crest-derived eYFP+ cells relative to total cells was significantly increased in Smad2nc-/- corneas (Figure 2B). To assess whether this expansion resulted from active cell proliferation, we performed EdU incorporation assays. In control corneas, eYFP+ cells were largely quiescent, showing negligible EdU uptake. In contrast, Smad2nc-/- corneas exhibited extensive cell proliferation, with EdU signals strictly co-localizing with eYFP+ populations (Figure 2C). The proliferation rate in neural-crest-derived cells increased dramatically from nearly negligible in controls to approximately 60% in Smad2nc-/- mice (Figure 2D). Consistent with this hyper-proliferative state, marked upregulation of cell cycle regulators Cyclin D1 and PCNA was observed in Smad2nc-/- corneas compared to the controls (Figure 2E). These findings demonstrate that Smad2 deficiency triggers a hyper-proliferative response in corneal cells.

### 3.3. Smad2 Deficiency Drives Severe Corneal Fibrosis and Aberrant ECM Accumulation

**Figure 3:**
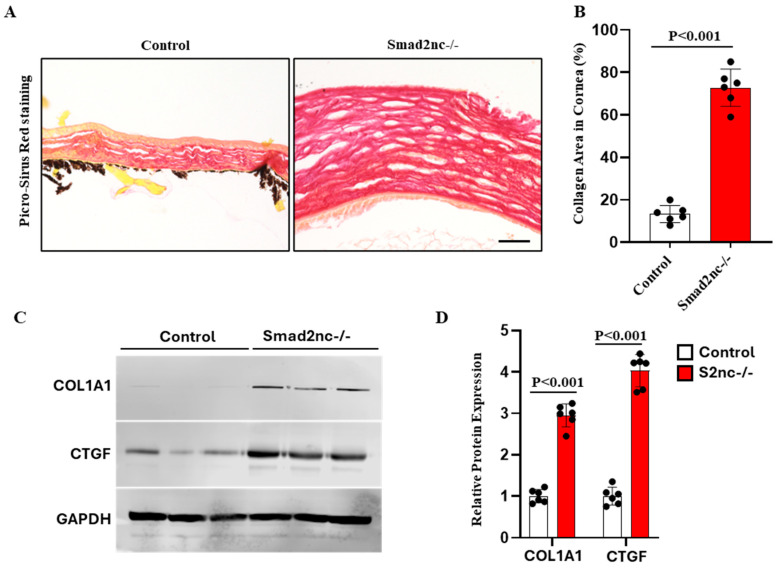
Smad2 deficiency caused severe corneal fibrosis and aberrant extracellular matrix expression. (**A**) Representative Picro-Sirius Red staining of corneal sections from Control and Smad2 neural-crest-specific knockout (Smad2nc-/-) mice. Wild-type (Control) corneas displayed a thin and compact stroma, whereas Smad2nc-/- corneas exhibited severe stromal thickening with massive collagen deposition. Collagen fibers displayed mixed birefringence under polarized light, with a color shifting from yellow to orange-red. Scale bar: 25 µm. (**B**) Quantification of the collagen-positive area in the cornea. The percentage of fibrotic areas significantly increased in the Smad2nc-/- group (~70%) compared to controls (~15%). Data are presented as mean ± SD. *p* < 0.001 (Student’s t-test), n = 6. (**C**) Western blot analysis of corneal lysates validating the expression of key profibrotic mediators. COL1A1 (Collagen Type I Alpha 1) and CTGF (Connective Tissue Growth Factor) were markedly upregulated in Smad2nc-/- corneas compared to the controls. GAPDH was used as a loading control. (**D**) Quantification of relative protein expression. Both COL1A1 and CTGF were upregulated in the absence of Smad2. Data are presented as mean ± SD. *p* < 0.001 (Student’s t-test), n = 6.

To further characterize the structural basis of corneal opacity, we assessed collagen deposition using Picro-Sirius Red staining. Control corneas displayed thin and compact stroma with regularly arranged collagen fibers. In contrast, Smad2nc-/- corneas exhibited intense and diffuse red staining and significant stromal thickening, indicating massive and disorganized collagen deposition (Figure 3A). There were also significantly increased fibrotic areas in Smad2nc-/- corneas (~70%) compared to controls (~15%) (Figure 3B). At the molecular level, the expression of key profibrotic mediators, i.e., collagen type 1α (COL1A1) and connective tissue growth factor (CTGF), was markedly upregulated in Smad2nc-/- corneas compared to the controls (Figure 3C). Densitometric quantification confirmed a greater than three-fold induction of both proteins in the absence of Smad2 (Figure 3D), confirming the activation of a robust fibrotic program.

### 3.4. In Silico Perturbation Suggests Smad3, Tead2, and Yap1 as Potential Regulatory Targets of Smad2

**Figure 4:**
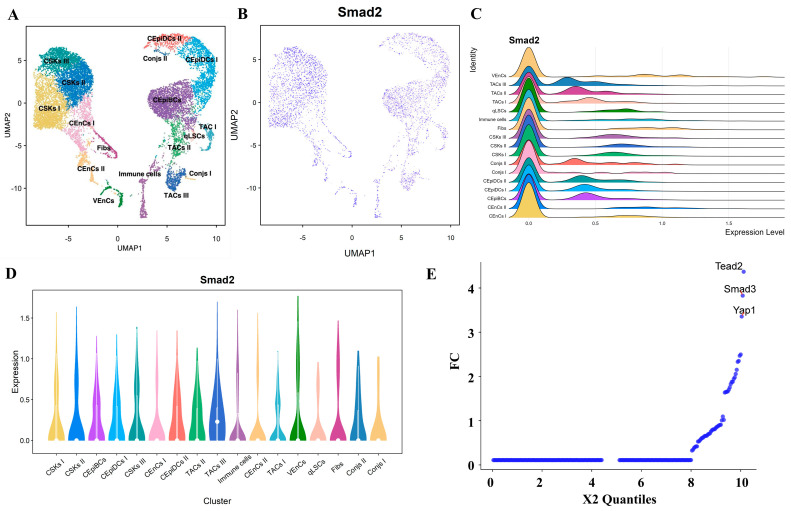
Single-cell RNA sequencing and in silico perturbation analysis indicated Smad3, Tead2, and Yap1 as regulatory targets of Smad2 in corneal keratocytes. (**A**) Uniform Manifold Approximation and Projection (UMAP) visualization of 14,735 single cells from the mouse cornea (Dataset PRJNA891516). Unsupervised clustering identified distinct cell populations annotated based on canonical marker expression. (**B**–**D**) Characterization of Smad2 expression across the corneal landscape. (**B**) Feature plot, (**C**) Ridge plot, and (**D**) Violin plot showed that Smad2 is broadly expressed but exhibits robust levels in neural-crest-derived stromal compartment (CSKs and Fibs), supporting these cells as the primary responders in the Wnt1-Cre-mediated knockout model. (**E**) In silico analysis of Smad2 knockout using the scTenifoldKnk algorithm. The scatter plot ranked genes based on the deviation in their regulatory network stability (Manifold Alignment) following the virtual deletion of Smad2. Tead2, Smad3, and Yap1 were the top genes with the most significantly disrupted regulatory networks (highest perturbation scores), suggesting that they are the potential downstream effectors unleashed by Smad2 loss.

To elucidate the molecular network governing this phenotype, we analyzed single-cell RNA sequencing (scRNA-seq) data from the mouse cornea (Dataset PRJNA891516). Unsupervised clustering annotated based on canonical markers identified distinct cell populations, including *Kera*+ CSKs, *Col8a1*+ endothelial cells and *Krt12*+ epithelial cells (Figure 4A). Mapping of Smad2 expression across these clusters revealed robust levels within the neural-crest-derived stromal compartment (Figure 4B–D), suggesting these cells as primary responders to Wnt1-Cre-mediated Smad2 deletion. We thus performed an in silico knockout analysis using the scTenifoldKnk algorithm to predict the impact of *Smad2* loss on the GRN. This virtual deletion suggested that *Tead2*, *Smad3*, and *Yap1* are top candidate genes with the most significantly disrupted regulatory stability (Figure 4E), suggesting that Smad2 removal could unleash a pathogenic Smad3/Tead2/Yap1 axis in corneal stromal cells.

### 3.5. Smad2 Deficiency Facilitates the Assembly of Pathogenic Nuclear Smad3/YAP/TEAD2 Complex

**Figure 5:**
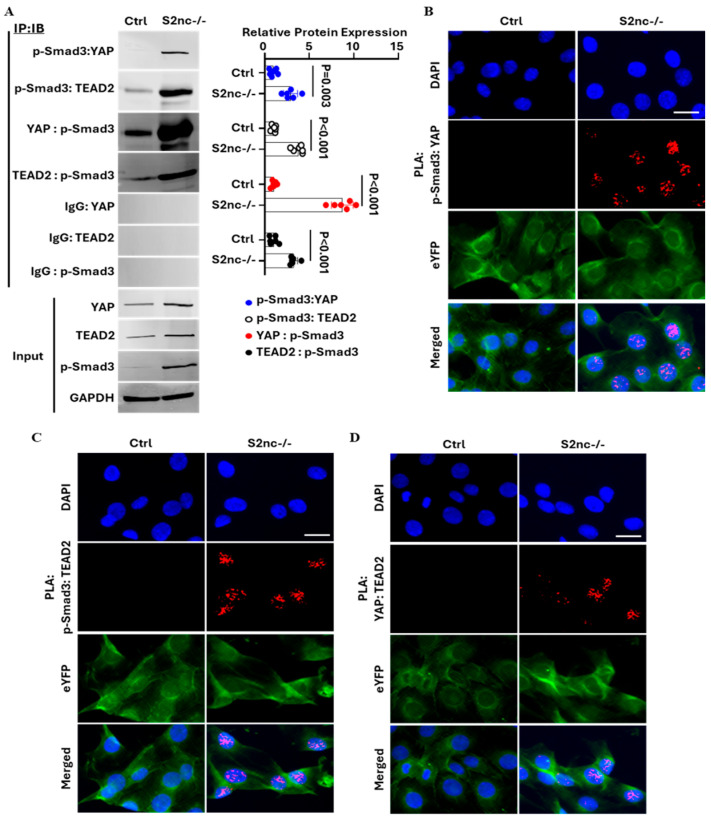
Smad2 deficiency in neural crest cells (S2nc-/-) facilitated the assembly of p-Smad3/YAP/TEAD2 transcriptional complex in the nuclei of mouse corneal cells. (**A**) Co-Immunoprecipitation (Co-IP) assay and Western blot analysis of corneal stromal lysates from wild-type (Ctrl) and S2nc-/- mice. Top panels (IP): Co-IP of p-Smad3 successfully pulled down both YAP and TEAD2 in S2nc-/- corneal lysates but not in WT corneas. Reciprocal Co-IP of TEAD2 or YAP pulled down p-Smad3. Bottom panels (Input) showed that the expression levels of TEAD2, YAP, and p-Smad3 were markedly upregulated in S2nc-/- corneas compared to WT (GAPDH used as loading control). Co-IP protein levels were quantified by normalized to the corresponding protein input signal and analyzed with unpaired two-tailed Student’s t-test. Data presented as mean ± SD. (**B**–**D**) In situ detection of protein–protein interactions using Proximity Ligation Assay (PLA) in corneal flat mounts. Neural-crest-derived cells were marked by eYFP (green), and nuclei were stained with DAPI (blue). Red fluorescent puncta indicate positive interaction (<40 nm proximity). (**B**) PLA indicating interaction of Smad3 with YAP. S2nc-/- cells displayed profound formation of Smad3/YAP complexes specifically in the nuclei, while Ctrl cells showed no signal. (**C**) PLA indicating p-Smad3–TEAD2 interaction. Abundant nuclear red puncta in S2nc-/- cells confirmed that Smad3 was directly bound to TEAD2. (**D**) PLA indicating YAP–TEAD2 interaction. The extensive nuclear signal in S2nc-/- cells demonstrates the assembly of the YAP/TEAD transcriptional machinery. Scale bar: 10 µm for all panels.

To validate the in silico predictions, we investigated the effects of Smad2 on the interactions of these candidate proteins. Co-Immunoprecipitation (Co-IP) assays with corneal stromal lysates demonstrated that immunoprecipitation of p-Smad3 pulled down significant levels of both YAP and TEAD2 in Smad2nc-/- lysates, which is much less in control corneas (Figure 5A), suggesting that Smad2nc-/- promoted the formation of p-Smad3/YAP/TEAD2 complex. Reciprocal IPs confirmed this physical complex. Furthermore, total protein levels of TEAD2, YAP, and p-Smad3 were markedly upregulated in the absence of Smad2 (Figure 5A), suggesting that the enhanced Co-IP signals are likely due to, at least in part, the increased abundance and nuclear availability of these proteins. We also visualized these interactions in situ using Proximity Ligation Assays (PLA). While WT cells showed no signal, Smad2nc-/- keratocytes displayed abundant nuclear red puncta indicative of p-Smad3-YAP (Figure 5B), p-Smad3-TEAD2 (Figure 5C), and YAP-TEAD2 interactions (Figure 5D). Consistently, immunostaining showed the robust nuclear accumulation of p-Smad3, TEAD2, and YAP in Smad2nc-/- cells, contrasting with the weak or cytoplasmic signals seen in controls (Supplementary Figure S1A–C). These data indicate that Smad2nc-/- promotes the formation of Smad3/YAP/TEAD2 complex by enhancing their expression and/or increasing their interaction capacity.

### 3.6. Smad3-Dependent Activation of YAP/TEAD Program Drives Fibrotic Gene Promoter Activities

**Figure 6:**
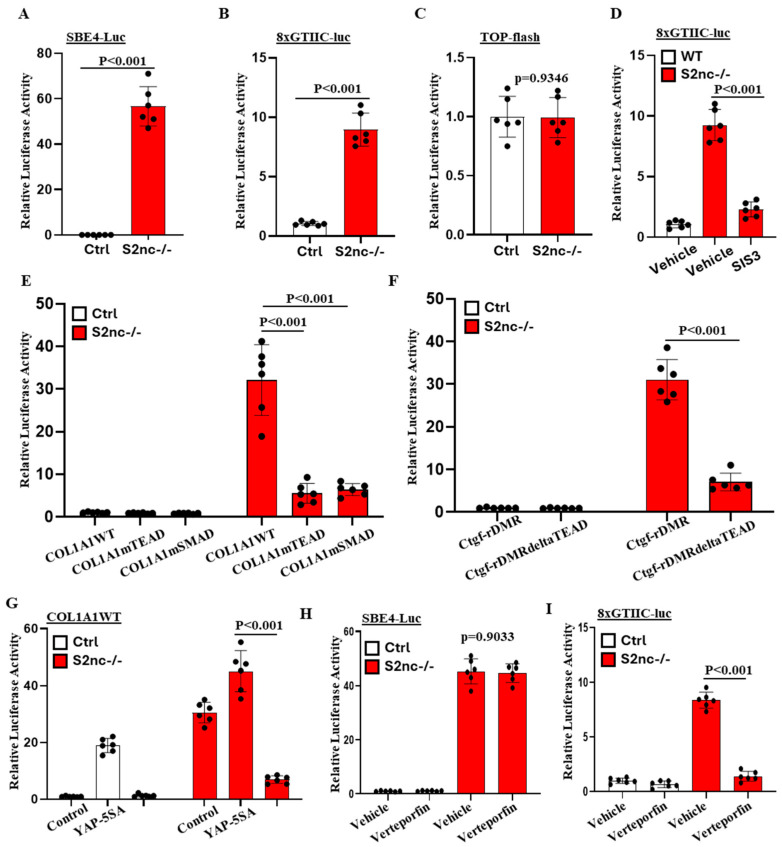
Smad2 deficiency promoted fibrotic gene expression via Smad3-dependent activation of the YAP/TEAD transcriptional program. (**A**–**C**) Analysis of pathway-specific transcriptional activity in primary keratocytes using luciferase reporter assays. (**A**) The Smad reporter (SBE4-Luc) showed markedly elevated activity in Smad2 neural-crest-deficient (S2nc-/-) cells compared to wild-type cells (Ctrl). (**B**) The YAP/TEAD reporter (8×GTIIC-luc) was significantly upregulated in S2nc-/- cells. (**C**) The Wnt reporter (TOP-flash) showed no significant difference between groups, indicating pathway specificity. (**D**) Hierarchical validation using a specific inhibitor. S2nc-/- cells were transfected with 8×GTIIC-luc reporter and treated with the Smad3-specific inhibitor SIS3 (or vehicle control). Inhibition of Smad3 activity significantly attenuated the aberrant YAP/TEAD reporter activity, demonstrating that the hyperactive signaling was driven by Smad3 activation. (**E**,**F**) Promoter analysis of fibrotic target genes. (**E**) Col1a1 promoter (COL1A1WT) activity was drastically induced in S2nc-/- cells. Mutation of either the TEAD (COL1A1mTEAD) or the Smad binding site (COL1A1mSMAD) significantly blunted induction, demonstrating the importance of both factors. (**F**) Upregulation of the Ctgf promoter (Ctgf-rDMR) in S2nc-/- cells was completely abolished by TEAD-binding site mutation (deltaTEAD), identifying TEAD as an obligatory cofactor for Ctgf expression. (**G**) Constitutively active YAP (YAP-5SA) robustly promoted Col1a1 promoter activity, which was diminished by YAP with TEAD-binding defective mutation (YAP-5SA/S94A), confirming that the fibrotic transcriptional program was dependent on the physical interaction between YAP and TEAD. Data are presented as mean ± SD, n = 6. (**H**,**I**) YAP/TEAD inhibitor verteporfin blocked S2nc-/-activated YAP/TAZ-responsive promoter activity. (**H**) Smad3 reporter (SBE4-Luc) activity was unaffected by verteporfin, confirming that verteporfin did not off-target the upstream TGF-beta signaling. (**I**) YAP/TEAD-responsive promoter (8×GTIIC-luc) activity was drastically suppressed by verteporfin, suggesting that therapeutic rescue may be achieved by dismantling YAP-TEAD activity. Statistical analyses: two-tailed Student’s t test for (**A**–**C**) and one-way Anova with Tukey’s multiple comparison test for (**D**–**I**).

To functionally link Smad3/YAP/TEAD2 complex to Smad2nc-/-caused fibrotic phenotype in corneas, we performed luciferase reporter assays in corneal stromal keratocytes. Smad2nc-/- cells exhibited hyperactivation of both TGF-β signaling (SBE4-Luc; Figure 6A) and Hippo/YAP signaling (8×GTIIC-luc; Figure 6B), while Wnt signaling remained unaffected (TOPflash, Figure 6C), likely due to the lack of Smad3 binding site in TOPflash reporter or the lack of binding partners in corneal keratocytes required for Smad3 interaction with Wnt downstream mediator T-cell factor/lymphoid enhancer factor, the driver of TOPflash reporter activity. Importantly, treatment with the specific inhibitor for Smad3 SIS3 significantly attenuated aberrant 8×GTIIC activation (Figure 6D), indicating that Hippo pathway hyperactivation is downstream of Smad3 signaling. Furthermore, the promoters of fibrosis markers *Col1a1* and *Ctgf* were drastically activated in Smad2nc-/- cells (Figure 6E,F). However, mutation of either the TEAD- or Smad-binding site on the *Col1a1* promoter significantly blunted its induction, demonstrating the essential roles of both factors (Figure 6E). Similarly, *Ctgf* upregulation was strictly dependent on the TEAD binding site (Figure 6F). Moreover, the TEAD-binding defective YAP mutant (*YAP-5SA/S94A*) failed to sustain *Col1a1* promoter activity compared to the functional constitutively active YAP (YAP-5SA, Figure 6G), confirming that the fibrotic program relies on the physical YAP–TEAD interaction. Finally, to validate the significance of YAP–TEAD interaction, we treated the corneal cells with YAP–TEAD disruptor verteporfin and found that verteporfin drastically suppressed YAP/TEAD-responsive promoter (8×GTIIC-luc) activity but did not affect the upstream Smad3 reporter activity in Smad2nc-/- cells (Figure 6H,I), further demonstrating that YAP/TEAD interaction is crucial for Smad2nc-/-mediated pathology.

### 3.7. Pharmacological Inhibition of YAP/TEAD Reverses Corneal Fibrosis and Restores Transparency

**Figure 7:**
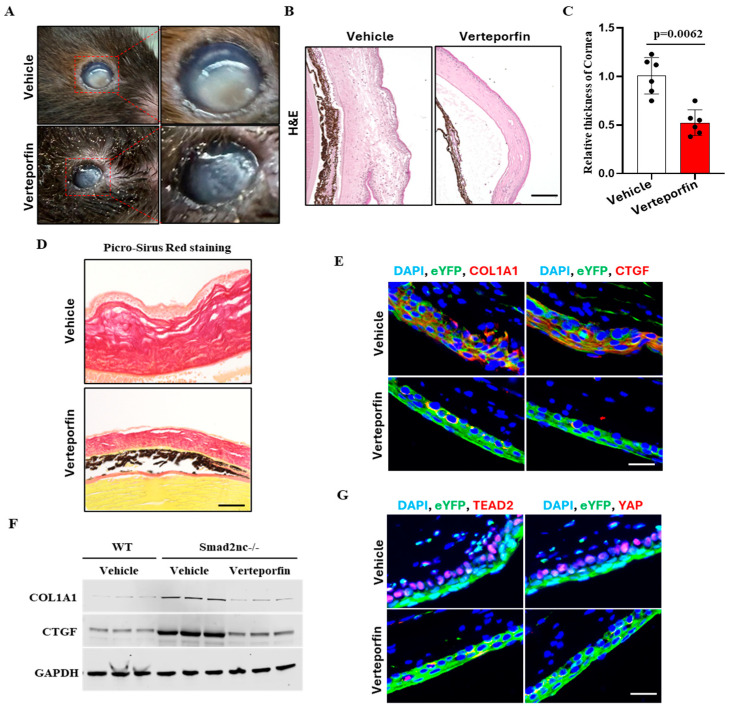
Pharmacological inhibition of YAP/TEAD signaling with verteporfin restored corneal structure and blocked corneal fibrosis in Smad2 neural-crest-deficient mice. (**A**) Representative gross eye images of Smad2 neural crest knockout (Smad2nc-/-) mice treated with vehicle or verteporfin. Verteporfin treatment made the corneas clearer and more transparent compared to vehicle-treated eyes. Insets show higher magnification of the central cornea. (**B**) Histological analysis (H&E staining) of corneal sections. Vehicle-treated corneas exhibited massive stromal hyperplasia and thickening. In contrast, VP-treated corneas displayed a normalized stromal architecture with significantly reduced cellularity and thickness. Scale bar: 30 µm. (**C**) Quantification of relative corneal thickness. Verteporfin treatment significantly reduced corneal thickness compared to the vehicle group. Data represent mean ± SD, Student’s t-test; n = 6. (**D**) Representative Picro-Sirius Red staining of corneal sections. The intense red staining indicative of excessive collagen deposition in vehicle-treated corneas was markedly reduced by verteporfin treatment. Scale bar: 30 µm. (**E**) Immunostaining of COL1A1 and CTGF (red) in Smad2nc-/- corneas. Neural-crest-derived cells were marked by eYFP (green). Verteporfin potently suppresses the expression of profibrotic proteins shown in the vehicle-treated corneas. Scale bar: 15 µm. (**F**) Western Blot analysis of corneal lysates confirming the molecular efficacy of verteporfin in COL1A1 and CTGF protein expression in Smad2nc-/- corneas. (**G**) Immunostaining of TEAD2 and YAP in Smad2nc-/- corneas. TEAD2 and YAP (Red) were highly expressed and located in the nuclei of vehicle-treated corneal cells but were significantly diminished in verteporfin-treated groups. Scale bar: 15 µm.

To test if corneal opacification and the underlying pathology can be reversed, we evaluated the therapeutic potential of verteporfin. Topical administration of verteporfin to Smad2-nc-/- mouse corneas on postnatal day 22 (after weaning) resulted in remarkable attenuation of the ocular phenotype at 3 months of age. While vehicle-treated eyes developed severe corneal opacity and plaque formation, verteporfin treatment significantly diminished the phenotype, resulting in clearer and more transparent corneas (Figure 7A). Histologically, verteporfin treatment normalized the stromal architecture, significantly reducing corneal thickness compared to the vehicle controls (Figure 7B,C). In addition, verteporfin treatment also eliminated fibrosis in Smad2nc-/- corneas (Figure 7D). At the molecular level, verteporfin potently suppressed the expression of fibrosis markers COL1A1 and CTGF, as shown by immunostaining (Figure 7E) and Western blot analysis (Figure 7F). Immunostaining also revealed that verteporfin treatment successfully inhibited the aberrant expression of TEAD2 and YAP proteins from the corneal cells (Figure 7G).

### 3.8. Verteporfin Ameliorates Hyper-Proliferation in Smad2-Deficient Corneas

**Figure 8:**
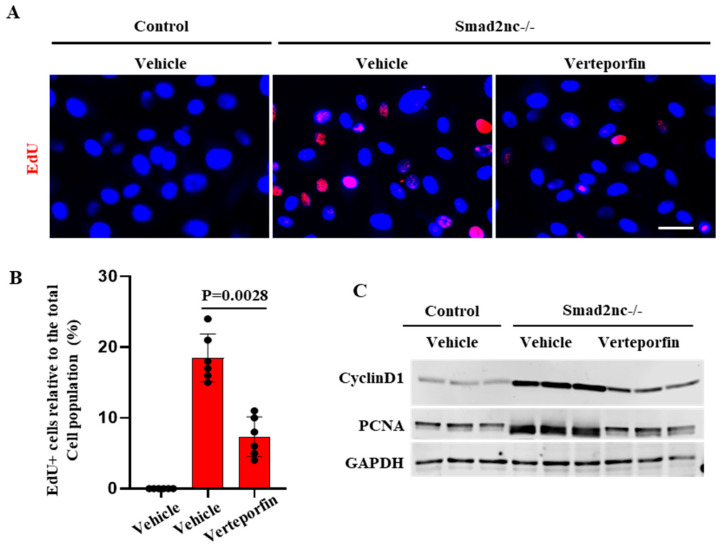
Verteporfin treatment suppressed aberrant cell proliferation in Smad2nc-/- corneas. (**A**) Representative immunofluorescence images of EdU incorporation (red) in corneal flat mounts. Smad2 neural crest knockout (Smad2nc-/-) corneas treated with vehicle exhibited extensive cell proliferation, as shown by EdU staining. YAP/TEAD inhibitor verteporfin treatment effectively suppressed cell proliferation, restoring a phenotype resembling the quiescent wild type (Control) corneas. Nuclei are stained with DAPI (blue). Scale bar = 25 µm. (**B**) Quantification of EdU-positive cells relative to the total cell numbers. Data represent mean ± SD. One-way Anova with Tukey’s multiple comparison test. (**C**) Western blot analysis of corneal lysates confirming the molecular efficacy of verteporfin. Verteporfin treatment drastically diminished Cyclin D1 and PCNA protein levels that are elevated in Smad2nc-/- corneas.

Because Smad2nc-/- caused hyperproliferation of corneal stromal cells, we investigated whether disrupting the YAP/TEAD complex could also reverse corneal cell proliferation. Indeed, VP treatment effectively suppressed aberrant EdU incorporation in Smad2nc-/- corneas, restoring a quiescent phenotype resembling the wild-type control corneas (Figure 8A). A significant reduction in the percentage of EdU+ cells was observed following treatment (Figure 8B). Consistently, Western blot analysis of corneal lysates showed that verteporfin treatment drastically reduced the protein levels of cell cycle regulators Cyclin D1 and PCNA, which were elevated in vehicle-treated Smad2nc-/- corneas (Figure 8C). These results indicate that blocking p-Smad3/YAP/TEAD complex by verteporfin can pharmacologically intercept both corneal cell proliferation and fibrogenesis, leading to the prevention of the development of corneal opacification due to Smad2 deficiency.

## 4. Discussion

Corneas rely on quiescence stromal cells to preserve transparency. In this study, we found that Smad2 deficiency in neural-crest-derived corneal cells triggers a spontaneous fibrotic and hyper-proliferative phenotype, leading to corneal opacity, resembling corneal stromal hypertrophy. Mechanistically, in the absence of Smad2, Smad3 nucleates a pathogenic trimeric complex with YAP and TEAD2. This complex directly binds to the promoters of cell cycle genes (*Ccnd1*) and fibrosis genes (*Col1a1*, *Ctgf*), driving the disease phenotype.

Smad2 loss destabilizing the regulatory networks of Smad3/YAP/TEAD2 was predicted by the in silico perturbation analysis, and their physical interactions were validated in Smad2nc-/- corneal cells. Notably, TEAD2, rather than the more ubiquitous TEAD1/4, was identified as a key component up-regulated in corneas. The requirement for both Smad3 and TEAD binding to the *Col1a1* promoter suggests that both TGF-β (p-Smad3) and Hippo signaling (YAP/TEAD) are involved in corneal fibrogenesis.

The functional distinction between Smad2 and Smad3 proteins has been a subject of long-standing debate. Our findings align with a growing body of evidence suggesting divergent roles of Smad2 and Smad3 in fibrogenesis. Smad2 buffers Smad3 activity to inhibit Smad3 fibrogenic activity in different organs, such as the kidneys and corneas. The increase in Smad3 activation upon Smad2 deletion (Figure 4 and Supplementary Figure S1) supports a competition model where Smad2 limits Smad3 activation and thus its access to fibrotic gene promoters. In addition, Smad2 seems to further block fibrosis specifically in corneas by disrupting the formation of Smad3/YAP/TEAD2 complex. In Smad2nc-/- corneas, increased Smad3 signaling occurs together with enhanced YAP/TEAD activity, which enhances the transcriptional environment for fibrotic gene expression. The spontaneous nature of the opacity in Smad2nc-/- mice suggests that Smad2 is critically important for maintaining corneal homeostasis and for preventing corneas from the runaway fibrotic response.

Interestingly, Smad3 and YAP/TEAD regulations appear not to be reciprocal. SIS3 suppression of the elevated YAP/TEAD reporter activity in corneal cells suggests that Smad3 regulates the YAP/TEAD transcriptional program. However, because inhibition of YAP/TEAD activity by verteporfin does not affect SBE4-Luc activity, YAP/TEAD is unlikely to reciprocally regulate Smad3 activity at this transcriptional level in corneal cells. Nevertheless, YAP/TEAD could modulate Smad3-dependent responses through different mechanisms, such as chromatin accessibility, nuclear retention, or target gene selection, which may be studied in the future.

The translational relevance of the Smad3/YAP/TEAD pathway is supported by prior studies implicating TGF-β/Smad and YAP/TAZ/TEAD signaling in corneal stromal activation and fibrosis. In primary human corneal fibroblasts, YAP and TAZ have been shown to regulate TGF-β1-induced myofibroblast transformation and profibrotic gene expression [27], which suggests that Smad/YAP/TEAD crosstalk may contribute to human corneal scarring or postoperative haze. However, further studies are required to determine whether the specific Smad2-deficiency-driven Smad3/YAP/TEAD2 mechanism is active in human corneal disease by using human corneal scar specimens or detecting the genetic relevance of Smad2 to corneal opacity. Thus, the verteporfin treatment approach used in this study may be considered as preclinical proof of concept for local YAP/TEAD inhibition rather than a clinical treatment regimen.

On the other hand, verteporfin has already been used clinically in ophthalmology as a photosensitizer for photodynamic therapy, but approved ocular use involves intravenous administration followed by local light activation. Our study using repeated topical application in a hyaluronic-acid-based gel without photodynamic activation may be beneficial for corneal disease because it limits systemic exposure. However, proper formulation of drug and pharmacologic studies, such as ocular surface retention, stromal penetration, and/or target engagement, are necessary before clinical translation. Recent preclinical work using verteporfin/hyaluronic acid gel supports the feasibility of local ocular delivery for reducing corneal scarring, but dose–response relationships, pharmacokinetics, long-term ocular safety, and optimal treatment windows remain to be defined. Moreover, YAP/TEAD inhibition may be applicable as an early-disease-modifying, scar-preventive, or transplant-sparing strategy after corneal injury, infection, or surgery, especially for patients with YAP/TEAD-dependent transcription remaining targetable. Identifying these patients is crucial although challenging because established acellular scar tissue may be less responsive than actively remodeling corneal stroma. Thus, verteporfin treatment may not replace corneal transplantation for patients with advanced, irreversible opacity, severe stromal disorganization, endothelial failure, or structural tissue loss.

Verteporfin-treatment-related ocular toxicity is not obvious with gross inspection, although a few mice showed body weight loss. It is unclear whether weight loss is due to verteporfin exposure, ocular disease burden, or other nonspecific factors, which warranties future investigation of the potential side effects of verteporfin, such as epithelial toxicity, endothelial toxicity, photosensitivity, and systemic absorption. Moreover, while verteporfin is widely used to disrupt YAP/TEAD-dependent transcription, it may also have YAP/TEAD-independent effects, such as photosensitizer activity, cellular stress responses, or effects on protein homeostasis. Future studies using more selective TEAD inhibitors and/or genetic YAP/TEAD loss-of-function approaches may determine whether off-target mechanisms are involved in the observed effects of verteporfin.

Our studies have some limitations. Although Wnt1-Cre is widely used to target neural-crest-derived cells, it is not specific to corneal cells. There are also other caveats with Wnt1-Cre-mediated recombination, including broad neural crest lineage labeling, germline leakage, and potential developmental effects associated with the Wnt1-Cre transgene itself [37,38]. Therefore, the phenotype observed in Smad2nc-/- mice may not be only attributable to neural-crest-derived corneal cells but also other non-neural crest-derived cells. Future studies using a better and inducible neural crest cell Cre line, such as Sox10-CreERT2 mice, inducible corneal cell-specific Cre drivers, and/or corneal transplantation approaches may determine if and to what extent Smad2 loss drives the Smad3/YAP/TEAD-dependent fibrotic pathway in neural-crest-derived corneal cells or if other cells are also involved.

In summary, our findings support a model in which Smad2 contributes to corneal stromal homeostasis by limiting excessive activation of Smad3-associated profibrotic signaling and YAP/TEAD-dependent transcription. Smad2 deficiency increases Smad3 signaling coinciding with nuclear accumulation of YAP and TEAD2 and enhanced formation of Smad3/YAP/TEAD2 complexes and further activates fibrotic and proliferative gene programs, leading to corneal opacity. Importantly, targeting YAP/TEAD2 using verteporfin blocks Smad2 deficiency-caused corneal opacity, suggesting that Smad3/YAP/TEAD2 is a targetable pathway for a future therapeutic invention to treat corneal opacity.

## Data Availability

The published single-cell RNA sequencing dataset analyzed in this study is available through NCBI BioProject under accession number PRJNA891516. No new high-throughput sequencing dataset was generated in this study. The original contributions presented in the study are included in the article/Supplementary Materials; further inquiries can be directed to the corresponding author.

## Supplementary Materials

The following supporting information can be downloaded at https://www.mdpi.com/article/10.3390/cells15131202/s1. Figure S1: Loss of Smad2 triggers nuclear accumulation and upregulation of the pathogenic triad: p-Smad3, TEAD2, and YAP. Table S1: Primary antibodies used for immunostaining and immunoblotting.
