# Supplementary material for: Smad2 Preserves Corneal Stromal Homeostasis by Restraining Profibrotic Smad3/YAP/TEAD2 Transcriptional Program
Source: Cells. 2026 Jul 2;15(13):1202. doi: 10.3390/cells15131202 (PMC13360062; doi:10.3390/cells15131202)
Supplement: Supplementary file 1 [file cells-15-01202-s001.zip › cells-4241671-supplementary.pdf]

Smad2 preserves corneal stromal homeostasis by restraining pro-fibrotic Smad3/ YAP/TEAD2 transcriptional program

Ruimei Zhou[1,2], Dunpeng Cai[1], & Shi-You Chen[1,3]*

[1]Departments of Surgery, University of Missouri School of Medicine, Columbia, MO; USA

[2]Departments of Medical Pharmacology and Physiology, University of Missouri School of Medicine, Columbia, MO; USA

[3]The Research Service, Harry S. Truman Memorial Veterans Hospital, Columbia, MO, USA

Supplemental Materials

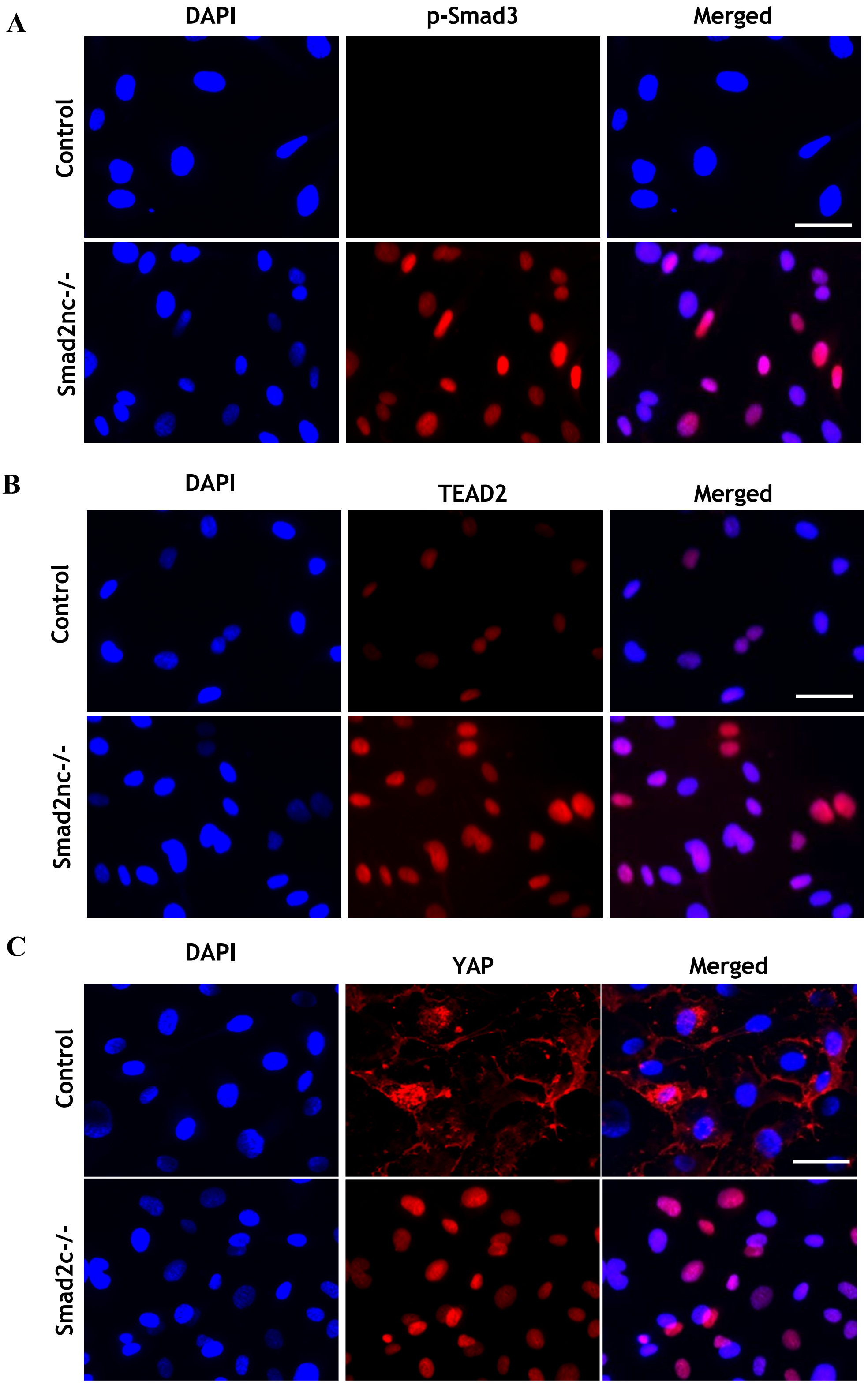

Supplement Figure S1. Loss of Smad2 triggers nuclear accumulation and upregulation of p-Smad3, TEAD2, and YAP. A, Representative immunostaining for phospho-Smad3 (p-Smad3) (red) in corneal sections from wild-type (Control) and Smad2 neural crest knockout (Smad2nc-/-) mice. Nuclei are counterstained with DAPI (blue). In control corneas, p-Smad3 signal is negligible, consistent with the quiescent state. In contrast, Smad2nc-/- corneas exhibit robust nuclear accumulation of p-Smad3, indicating TGF-β/Smad3 pathway activation. **B**, Immunostaining of TEAD2 expression (red). While control keratocytes show weak nuclear TEAD2 signal, Smad2nc-/- cells display significantly enhanced nuclear fluorescent intensity, confirming the upregulation of Tead2 predicted by in-silico Smad2 knockout analysis. **C**, Assessment of YAP subcellular localization (red). Control keratocytes exhibit predominant cytoplasmic retention of YAP (inactive), appearing as perinuclear rings. Smad2nc-/- leads to a sharp shift of YAP translocation into nuclei (active form). Scale Bar: 10 μm.

Supplemental Table S1. Primary antibodies used for immunostaining and immunoblotting.

Target	Host	Manufacturer	Cat number
GFP/eYFP	Rabbit	Invitrogen/Thermo Fisher	Cat. A-11122
p-Smad3 Ser423/425	Rabbit	CST	C25A9 Rabbit mAb #9520
CTGF / CCN2	Rabbit	CST	D8Z8U Rabbit mAb #86641
YAP	Mouse	CST	mAb #12395
COL1A1/Collagen I	Rabbit	Abcam	Cat. ab21286.
Cyclin D1	Rabbit	CST	92G2 Rabbit mAb #2978
PCNA	Rabbit	CST	D3H8P Rabbit mAb #13110
GAPDH	Rabbit	ProteinTech	Cat No. 10494-1-AP
TEAD2	Rabbit	Invitrogen/Thermo Fisher	Cat. 21159-1-AP
TEAD2	Mouse	Abcam	mAb clone 404C5a, Cat. ab54374.
